# Estimates of prevalence of anti-SARS-CoV-2 antibodies among blood donors in eight provinces of South Africa in November 2021

**DOI:** 10.21203/rs.3.rs-1359658/v1

**Published:** 2022-02-15

**Authors:** Russel Cable, Charl Coleman, Tanya Glatt, Eduard Grebe, Laurette Mhlanga, Cynthia Nyoni, Nadia Pieterson, Ronel Swanevelder, Avril Swarts, Wendy Sykes, Karin van den Berg, Marion Vermeulen, Alex Welte

**Affiliations:** 1 South African National Blood Service; 2 Western Cape Blood Service; 3 DSI-NRF Centre of Excellence in Epidemiological Modelling and Analysis (SACEMA), Stellenbosch University; 4 Vitalant Research Institute

**Keywords:** COVID-19, seroprevalence, anti-SARS-CoV-2 antibodies, blood donors, South Africa

## Abstract

In line with previous instalments of analysis from this ongoing study to monitor ‘Covid Seroprevalence’ among blood donors in South Africa, we report on analysis of 3395 samples obtained from 8 to 12 November 2021 in all provinces of South Africa except the Western Cape. As in our previous analyses, we see no evidence of age and sex dependence of prevalence, but substantial variation by province, and by race within each province, from which we generated provincial total point estimates (EC-74%; FS-75%; GP-68%; ZN-73%; LP-66; MP-73%; NC-63%; NW-81% ) and a ‘South Africa minus Western Cape’ national prevalence estimate of 71% (95%CI 69–74%). We note that sample collection occurred just before the omicron variant driven wave in South Africa, but otherwise present these results without significant interpretation.

## Introduction

We have previously published estimates of the prevalence of anti-SARS-CoV-2 antibodies among blood donors in South Africa, based on specimens collected from January to May 2021 [1,2], as well as estimates of fatality rates, based on these prevalence estimates and publicly available excess deaths estimates [3].

While the interpretation of seroprevalence is increasingly complicated by vaccination coverage and multiple infections of individuals, we understand that seroprevalence is still relevant from the point of view of understanding aspects of transmission and collective immunity that are relevant to ongoing adjustment of anti-transmission measures, policies, and regulation.

## Methods

Anticipating that a fourth wave late in 2021 was inevitable (though there was no knowledge of the omicron variant at the time of planning), 3395 specimens were randomly collected from consenting donors from all provinces except the Western Cape (which has its own separate blood service) presenting to donate blood to the South African National Blood Service (SANBS) from 8 to 12 November 2021. This was carried out in accordance with previous arrangements and practices underlying our previous rounds of sampling [1,2], as approved by the SANBS Human Research Ethics Committee (HREC).

For analysis, serology data was linked to basic donor demographic information (age, sex and race) but not to any other underlying data potentially available from the SANBS donor database (donation history, specific locale of donation/residence, donor identifiers etc).

Samples were tested for the presence of antibodies to SARS-CoV-2 nucleocapsid proteins, using the Roche Elecsys platform. These antibodies are typically developed in response to natural infection, but not in response to any vaccine currently available in South Africa. While there is some waning of antibodies, in this context we expect about 95 percent of donors who ever developed antibodies to have detectable levels at the time of testing [4] while a small proportion (we estimate in the range of 5–10 percent) of individuals who experience infection never develop detectable antibodies. [5]

## Results

**Figure 1:**
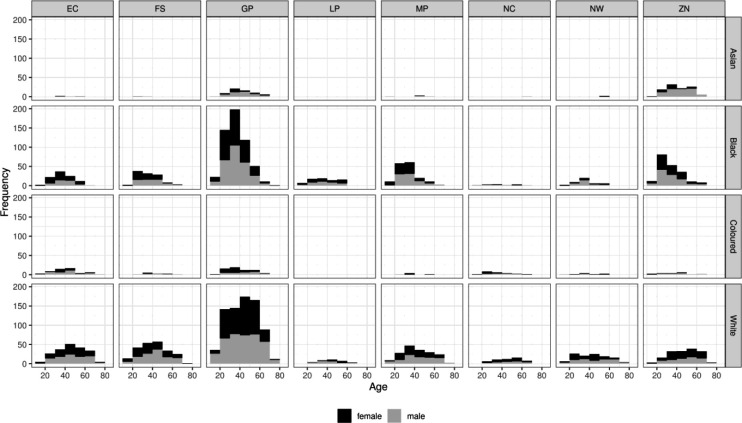
Age distribution of specimens included in the present analysis, further decomposed by race and province.

**Figure 2:**
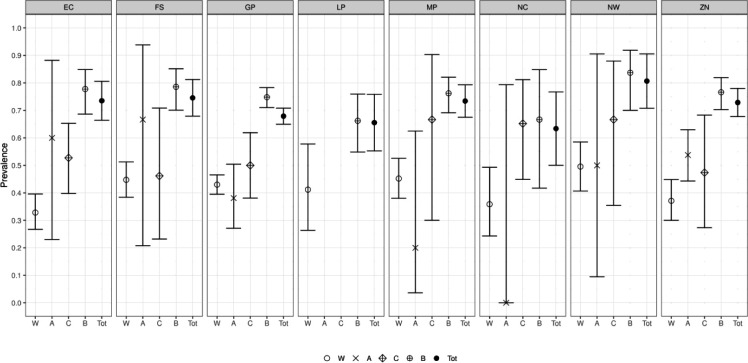
Seroprevalence estimates, for each sampled province, by race, and a race weighted provincial total.

Figure 1 shows the distribution of specimens by age group, race, sex and province. We found no statistically significant dependence of seroprevalence on either age or sex. However, unsurprisingly, there were statistically and epidemiologically significant differences between provinces, and among race groups in each province. Hence:

- We report primary results by every combination of province and racial group (Figure 1).
- From these, we generated a *race-weighted* seroprevalence for each province (Figure 2).
- From these provincial estimates, we generated a ‘national’ (minus Western Cape) estimate by weighting according to provincial population sizes (Table 1).

## Discussion

We note well known caveats about representativeness of donors, and also that at this stage of the epidemic there is an increasing incidence of reinfection. In this study we did not detect antibodies developed due to vaccination.

For the present purposes, we do not offer any substantial interpretation of these results, but trust that they will be of interest and use to modellers, policy makers and others.

More resource intensive surveillance, notably defined by:

- More frequent rounds of sampling
- Multi-assay testing algorithms to distinguish vaccine induced antibodies from antibodies produced by natural infection
- Sequencing

would potentially be significantly more informative.

## Figures and Tables

**Table 1:** The numerical provincial race-weighted seroprevalence values underlying figure 2

Province	Point Estimate (%)	95% Confidence Interval Lower bound (%)	95% Confidence Interval Upper bound (%)
Eastern Cape	73.5	66.0	81.0
Free state	74.6	67.5	81.6
Gauteng	67.9	64.9	70.9
Limpopo	65.6	54.6	76.5
Mpumalanga	73.4	67.2	79.6
Northern cape	63.4	48.1	78.7
North West	80.7	69.9	91.5
KwaZulu Natal	72.9	67.5	78.2
**8 Province Mean**	**71.1**	**68.8**	**73.5**
